# The dynamic hadronization of charm quarks in heavy-ion collisions

**DOI:** 10.1140/epjc/s10052-024-12593-0

**Published:** 2024-03-05

**Authors:** Christian Bierlich, Gösta Gustafson, Leif Lönnblad, Harsh Shah

**Affiliations:** 1 Department of Physics, Lund University, Sölvegatan 14A, 223 62 Lund, Sweden; 2 Institute of Nuclear Physics, Polish Academy of Sciences, Cracow, Poland

## Abstract

The Pythia8/Angantyr  model for heavy ion collisions was recently updated with a mechanism for *global colour reconnection*. The colour reconnection model used is QCD colour algebra inspired and enhances baryon production due to the formation of string junctions. In this paper, we present updates to the junction formation and string fragmentation mechanisms, connected to heavy quark fragmentation. This allows for the simulation of heavy quark fragmentation, using junction formation, in heavy ion collisions. The framework is validated for proton collisions, and we show results for charm baryon production in proton-lead collisions.

## Introduction

In high-energy particle collisions, hadrons with heavy quark content, are a uniquely versatile probe of fragmentation dynamics. Their defining feature, a charm ($\textrm{c}$) or bottom ($\textrm{b}$) flavoured quark, cannot originate from the hadronization process but must be created either in the hard process or in the parton shower, both calculable with perturbative techniques.

As opposed to the even heavier top ($\textrm{t}$) quark, hadrons containing $\textrm{c}$- and $\textrm{b}$-type quark content, are still understood to fragment through the same mechanisms as their light counterparts, the $\textrm{u}$, $\textrm{d}$, and $\textrm{s}$ quarks. When comparing experimental data to theory, two quite different (and thus complementary) techniques are used: The factorisation approach and the route taken by Monte Carlo event generators. In the factorisation approach [1, 2], the cross-section is separated into a convolution of three factors: (1) a Parton Distribution Function (PDF) of the incoming hadron, (2) the parton level hard scattering cross-section, where state-of-the-art calculations today are next-to-leading-order (NLO) in the strong coupling ($\alpha _s$) (see e.g. [3–5]) often with next-to-leading-log (NLL) resummation techniques applied as well, such as e.g. GM-VFNS [6] or FONLL [7, 8], and finally (3) fragmentation functions, analytical expressions fitted to $e^+e^-$ and $e\textrm{p}$ data [9, 10] giving differential probabilities for the charm quark to fragment to various hadron species. It has been known at least since SPS [11] that the underlying assumption of independent fragmentation does not hold, but it has generally been assumed that universal fragmentation functions can be applied across systems, when studying inclusive quantities, such as total charm hadron yields per event. Recent work by the ALICE collaboration [12–15] has, however, clearly shown that fragmentation functions tuned to $e^+e^-$ and $e\textrm{p}$, cannot describe the fragmentation of charm into baryons in $\textrm{pp}$.

In the Monte Carlo event generator approach, as used in e.g.  Pythia8  [16], PDFs are still used to extract the participating partons from the colliding nucleons. But where the focus in the factorisation approach tends to be more directed towards formal precision in the calculation of the hard scattering, the focus in the Monte Carlo generators is more towards coherent modelling of both perturbative and non-perturbative aspects, such as hadronization. Once the total amount of charm quarks present in the event is determined by means of a leading order calculation, plus parton shower [17, 18], the amount of hadron species, is determined by the dynamical fragmentation model, the Lund string model [19], and its extensions. This makes charm hadrons very well suited for studies of dynamical hadronization models. For charm baryon production in particular, the so-called QCD colour reconnection (CR) model [20] in Pythia8 has gained a lot of attention, due to its ability to correctly reproduce the $\Lambda ^+_c$ yield and $\Lambda ^+_{c}/D^0$ ratio as a function of $p_\perp$ in $\textrm{pp}$ collisions at various collision energies at LHC [12, 14, 15]. However, the predicted production rates of $\Xi _c$ and $\Omega _c$ baryons are still undershooting data, even with the QCDCR model [21–23]. Furthermore, the model has, until recently not been usable for heavy ion collisions.

One of the key aspects of the QCDCR model is the formation of junction-like configurations between two or three colour dipoles. These junction systems contribute to baryon production in addition to the baryons produced during the string fragmentation in Pythia8. We have recently improved the junction fragmentation for the low-energy junction systems and extended the QCDCR model with a spatial constraint [24]. As a result, the QCDCR model can be used as a global CR model for heavy-ion collision simulations in the Angantyr model [24, 25].

In this paper, we further improve the junction formation and fragmentation for the colour dipoles containing heavy quarks. We use $\textrm{pp}$ collisions to validate the framework and show for the first time how $\textrm{pPb}$ collisions generated with Angantyr + QCDCR, give a satisfactory description of $\Lambda ^+_c$ production. We show results primarily for charm baryons, but a similar outcome can be expected for the bottom quark containing baryons as well. We present the results using the upgraded QCDCR model from [24] with the new changes we have made in this paper.

We first provide an overview of the Angantyr model for heavy-ion event simulation in Pythia8 in the next section. In Sect. 3 we discuss the perturbative production of the charm quarks, and in Sect. 3.2, we show the non-perturbative aspects of the charm hadrons production. We also discuss the changes we have made in junction formation and fragmentation. Finally, results for charm hadron production in $\textrm{pp}$ and $\textrm{pPb}$ are shown in Sect. 4.

## Heavy ion collisions with the Angantyr model

The Angantyr model [25, 26] is an extension of Pythia8 to simulate heavy-ion collision events without assuming the creation of a Quark–Gluon plasma. It uses a modified Glauber model [27, 28] to obtain the number and types (e.g. elastic or inelastic (diffractive or non-diffractive) interactions) of sub-collisions in a heavy-ion collision event. Based on the number and type of sub-collisions, multiple $\textrm{pp}$-like collisions are generated and stacked together to produce the heavy-ion event.

The arrangement of the nucleons inside a nucleus is obtained using the Woods-Saxon distribution in the GLISSANDO parametrization [29]. When nuclei collide with each other at relativistic energies, they are Lorentz contracted. The wave functions of the nucleons inside the nuclei can be treated as frozen at the time of the collision. This is realized in the so-called Glauber–Gribov [30–32] formalism for nucleon wave-function fluctuations and extended it to include cross-section fluctuations in projectile and target nucleons for pA and AA collisions.

Once the types of nucleon–nucleon (NN) sub-collisions are decided, the Angantyr model uses the Pythia8 model for multiparton interactions to generate respectively non-diffractive, diffractive, and elastic $\textrm{pp}$ events. Often it occurs that a nucleon is participating in more than one NN non-diffractive sub-collision. A key feature of the model is the special treatment for nucleons participating in multiple non-diffractive interactions. Given a single projectile nucleon interacting with several target nucleons, the NN pair with the smallest impact parameter is denoted the “primary” non-diffractive sub-collision. The others are denoted “secondary”. The primary sub-collision is generated as a normal non-diffractive $\textrm{pp}$ collision, whereas the secondaries are generated as a modified single diffractive collision (see section 5 in Ref. [25] for further explanation). A secondary non-diffractive interaction will be discarded once sufficient energy is no longer available.

There is no interaction between the partons produced in different sub-collisions in the default Angantyr. All multiple sub-collisions are stacked together at the parton level as colour singlet Lund strings. Later, the Lund strings are hadronised and produce a heavy-ion collision event.

Recently we have added a global colour reconnection (CR) in Angantyr [24]. We have extended the QCDCR model [20], by adding a spatial constraint on the colour dipoles to be colour reconnected. We stack the colour dipoles from different sub-collisions and use the spatially constrained QCDCR model such that colour dipoles from nearby sub-collisions can undergo CR. In this work, we continue to use this upgraded Angantyr set-up to simulate $\textrm{pPb}$ collision events.

## Charm hadron production in Pythia8

Since the masses of charm ($\approx$1.5 GeV) and bottom ($\approx$4.8 GeV) are large compared to the light quarks, they will never be produced through the tunnelling mechanism by which the string breaks, but only in the hard process and the parton shower. In Sect. 3.1 we will briefly review the Pythia8 formalism for heavy quark production, and in Sect. 3.2 we give an overview of the impact of CR. In the following sub-sections, the modifications relevant to charm production in $\textrm{pPb}$ will be introduced.

### Charm quark in hard process and parton shower

Several different QCD processes in $\textrm{pp}$ collisions in Pythia8 can produce heavy quarks. The leading order (LO) processes like $qq \rightarrow Q\bar{Q}$ and $gg \rightarrow Q\bar{Q}$ hard scatterings are the primary processes for heavy quarks production in Pythia8. Another source of heavy quark production is weak decays (*Z* and $W^{\pm }$ bosons decays), Higgs decay, and top and bottom quark decay, though of those, only the latter contributes in any significant amount when considering total charm production down to low $p_\perp$. Furthermore, parton showers, where initial or final state partons (mostly gluons) produce the heavy quarks by pair creation, flavour excitation or gluon splitting. This is a significant source of charm production, in addition to that produced in the hard scattering. Furthermore, the “hidden charm” from the PDF of one of the colliding beams, may come on a mass shell due to the scattering. The interaction is like $Qq \rightarrow Qq$ or $Qg \rightarrow Qg$, but since the Q is not a valence quark it has to be produced in pairs by a gluon splitting.

The LO processes have the matrix elements containing the heavy quark mass. Since quark masses are included, full phase space down to $p_{\perp } \rightarrow 0$ can be populated. For low $p_\perp$ production, however, using the Pythia8 multiparton interaction framework [33], which introduces a general parameter $p_{\perp 0}$, is more suitable, in particular when extending to heavy ion collisions. The heavy quark masses are an important parameter in the perturbative description of their production. In Pythia8, the default values for the charm and bottom quark masses are set to 1.5 GeV and 4.8 GeV respectively. The masses affect the matrix elements, splitting kernels, and the phase space of the heavy quarks production cross-section. These values are fitted to *D*-meson production rates. To better fit production rates at LHC in [34] authors show that a reduced charm quark mass is expected. Following the arguments in [34] one can also expect a similar correction in the bottom quark mass. We have reduced the charm and bottom quark masses to 1.3 GeV and 4.2 GeV respectively in this paper.

### Colour reconnection and hadronization

After the multiple parton scatterings and parton showers, outgoing quarks and gluons are connected by strings. We speak of a string connecting a colour and an anti-colour – either quark and anti-quark or through one or more gluon “kinks” – as a chain of colour dipoles. These colour connections are reassigned through colour reconnection (CR) [20, 33] models. The conventional argument, and indeed the logic behind the default CR model in Pythia8, is that while the parton shower generates a colour configuration in the $N_{c}\rightarrow \infty$ limit, nature has $N_c = 3$. The choice of specific colour connections for a single event is ambiguous and should therefore be corrected. The calculation itself, however, cannot provide any guidance as to how to do the reconnection, and one must resort to models. A common feature is a reduction of the so-called $\lambda$-measure, which is an indirect representation of the rapidity span of the colour dipoles, which is again a logarithmic sum of the potential energy of the dipoles, and hence a measure of the number of hadrons produced by the dipoles. Further details in Sect. 3.3.1. The CR in Pythia8 helps to reproduce the charged particle multiplicity and the increase in $\langle p_\perp \rangle$ as a function of $(N_{ch})$ distribution as observed in the experiments.

**Fig. 1:**
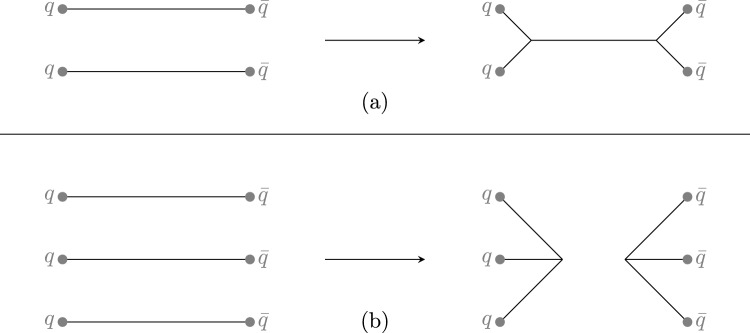
Illustration of colour reconnections forming junctions. Two dipoles can form a colour connected junction–anti-junction system (**a**), and three can form two separate (anti-) junction systems (**b**)

The QCDCR model [20] is developed with the idea of applying ${\textbf {SU(3)}}$ colour algebra on non-correlated colour dipoles before calculating the $\lambda$-measure for the new configurations of the dipoles. Colour algebra allows the formation of a colour singlet by three colour string pieces being connected to a “junction” point (see Fig. 1). The “string system contains a junction” (junction system) formed by two or three dipoles is not possible in the earlier case of $N_{c}\rightarrow \infty$ limit. Hence in the QCDCR model, the two and three dipoles can have three string pieces that are colour-connected to a single “junction” point after the CR. A junction system produces at least one baryon per junction during the hadronization. In the QCDCR model, junctions are always produced as junction and anti-junction pairs and conserve the baryon number. These baryons (and anti-baryons) are additional baryons due to QCDCR.

After the CR, the colour singlet Lund strings hadronised by sequential fragmentation. The different flavours of quarks and anti-quarks are produced according to the Lund string model [35]. Parameter values are fixed from the model tuning with LEP data [20, 36, 37].

The sequence of the string breaks decides if a string piece will form a meson or a baryon as a primary hadron. The Lund string fragmenting into $q \bar{q}$ pairs will produce mesons. For baryons production, the string has to break into a diquark–anti-diquark pair, where the consecutive string breaks of a $q \bar{q}$ pair on either side of the diquarks will produce a baryon and an anti-baryon. Pythia8 uses the “popcorn mechanism” [38], which includes a probability for a meson production between the baryon and the anti-baryon, and the results of the “popcorn mechanism” are supported by the experiments [39].

A $q\bar{q}$ pair production rate,

1 $$\begin{aligned} \textrm{d}P \simeq \textrm{d}^2p_{\perp } \exp \left( -\pi m^2_{\perp }/\kappa \right) , \hspace{4mm} m^2_\perp = p^2_\perp + m^2_{q}, \end{aligned}$$

where $\kappa$ is string tension, and $m_\perp$ is transverse mass of the quark with mass $m_{q}$ and opposite transverse momenta $p_\perp$. The pair production rate is mass-dependent, and it gives an extremely low probability for the production of heavy quarks pair (e.g. “charm” pairs) during the string fragmentation. Therefore all of the heavy quarks are produced either in hard scatterings or in parton showers in Pythia8 as mentioned in Sect. 3.1. In this paper, we show results for charm baryons only, so we refer to charm quarks as the heavy quarks for the rest of the paper.

The heavy quarks form mesons or baryons depending on which quarks/diquarks are produced next to them during string fragmentation. The only way the hadronization of the heavy quarks can be influenced is either by modifying fragmentation parameters or by colour reconnection. LEP data constrains the fragmentation parameters, while in the CR we have some freedom in rearranging the colour connections among the partons. Moreover, the QCDCR model allows junction configurations, which contribute to baryon production. In such a case the type of partons attached to the junction legs and the choice of junction fragmentation sequence can influence the baryon production. Moreover, the probability for junction formation increases in a more dense environment like high multiplicity $\textrm{pp}$ or heavy nuclei collisions.

Until now, no special attention was given if a heavy quark is involved during the junction formation or fragmentation. In this paper, we improve the junction treatments if one or more heavy quarks are involved. We discuss these improvements below in detail.

### The role of junctions

We have made several improvements in the QCDCR model in [24]. A crucial addition is the impact parameter-dependent constraint on the colour dipoles to be colour reconnected, which allowed us to have a global CR among the partons produced in different sub-collisions in heavy-ion event simulation in Angantyr. In addition, we implemented a few technical improvements in the hadronisation of junctions.

In Pythia8, there may occur situations when a string system in an event cannot be hadronised properly. There are several reasons for such failures, and often they involve junctions. If such a failure arises, Pythia8 will throw away the whole event and generate a new one. In $\textrm{pp}$ collisions such failures are typically rare, but in heavy-ion collisions there can be very many strings and the failure rate per event increases. And since the rate is higher for high multiplicity events (many strings), there is a risk that the overall multiplicity distributions may be skewed. With the QCDCR model, the number of junctions increases, which also increases the failure rate, and we found in [24] that the effect on multiplicity distributions was substantial in the heavy-ion collision, and even visible in $\textrm{pp}$ collisions.

A majority of the discarded events are found to have at least one junction system with a very low invariant mass ($\lesssim$1 GeV). We added a “junction collapse” mechanism to hadronize the low-mass junction systems, which were not treated in Pythia8 prior to [24]. This “junction collapse” mechanism produces two hadrons from the junction system. These hadrons can be two baryons or a meson and a baryon depending on the types of partons attached to the end of every leg in a junction system. We have also introduced an additional trial if the string fragmentation fails to hadronise a junction system. In such a scenario, the junction system is fragmented by the special version of the junction collapse procedure.

As we mentioned at the beginning of the paper, the conflicting results from $e^+e^-$ and $\textrm{pp}$ collisions raise questions about the universality of charm hadron production. In Pythia8, this applies in particular to charm baryons. We have seen that adding QCDCR improves the description for $\Lambda _c$, but for heavier charmed baryons there is still a problem. Since the additional junctions from the QCDCR model are responsible for the increased charm baryon rates, we want to look in more detail into how junctions involving charm quarks are handled there, and also how they are treated in the subsequent string fragmentation.

#### Junction formation

In $\textrm{pp}$ collisions, junctions are normally only formed in the treatment of the proton remnants, when more than one valence quark undergo scattering in the multiple parton interactions machinery, but such junctions mainly influence baryon production in the forward rapidity region. The junction formation due to colour reconnection is unique to the QCDCR model. The QCD colour algebra-based reconnection treatment includes the colour connections beyond the leading colour approximations. This means that besides the case where two uncorrelated dipoles having the exact same colour state can “swing” so that the coloured parton in one dipole becomes colour connected with the anti-colour of the other, and vice versa, there can also be reconnections between dipoles that have different colour states. In this way, the partons in two or three colour dipoles can become colour-connected to junction points (as shown in Fig. 1) with a certain probability that they are carrying the right colour charges. Each of the junction legs has to have a different colour charge so that the junction system becomes a colour singlet.

After all possible reconnections are tabulated, the QCDCR model will order them so that the reconnection reduces the string lengths the most, as defined by the $\lambda$-measure are performed first. For dipoles between two partons the $\lambda$-measure in the model is given by^1^

2 $$\begin{aligned} \lambda = \ln \left( 1 + \frac{\sqrt{2}E_1}{m_0} \right) + \ln \left( 1 + \frac{\sqrt{2}E_2}{m_0} \right) , \end{aligned}$$

where the energies $E_i$ are given in the dipole’s rest frame, and $m_0$ is a tunable parameter. For a dipole connecting a parton to a junction, the model similarly defines the $\lambda$-measure as

3 $$\begin{aligned} \lambda _j = \ln \left( 1 + \frac{\sqrt{2}E}{m_j} \right) , \end{aligned}$$

where *E* is the energy of the parton given in the junction rest frame^2^ and $m_j$ is a tunable parameter not necessarily the same as $m_0$.

As discussed in the introduction the $\lambda$-measure is an estimate of the rapidity range for the hadrons in the string breakup. The definitions in Eqs. (2 and 3) are well motivated for light quarks and massless gluons. However, for a string piece connected to a heavy quark, these expressions are not good estimates of the rapidity range. In this case, we instead use the rapidity of the heavy quark in the rest frame of the junction:

4 $$\begin{aligned} \lambda _{HQ} = \frac{1}{2} \log \left( \frac{E + p}{E - p} \right) . \end{aligned}$$

Here *E* and *p* are the energy and momentum of the heavy quark in the junction rest frame. The $\lambda _{HQ}$ will give a lower value than $\lambda _j$ for heavy quarks, especially for small *p*. Hence with this new change, we enhance the possibility for a heavy quark to be part of a junction system during CR in Pythia8. We note that there is no need for the parameter $m_j$ to set the scale in $\lambda _{HQ}$ since the quark mass does that for us. Also, the “1+” in the logarithm, which protects the $\lambda$ from becoming negative is also not needed.

#### Junction fragmentation

After the Colour Reconnections, the colour strings will undergo string fragmentation. In the QCDCR model in Pythia8, the three junction legs are treated separately according to the following steps.

- A few attempts are made to move the junction system to the junction rest frame.
- If the algorithm fails to obtain the junction rest frame, then the junction system fragments in the centre of the mass frame of the junction system.
- Once the frame is found, the summed energy of the partons on each junction leg is calculated in that frame and the junction legs are tagged as low-, middle-, and high-energy legs.
- The low-energy leg is fragmented first. A fictitious particle is assumed on the opposite side of the junction point for the given junction leg, and the string fragments from the endpoint towards the junction point until a parton closest to the junction point is left on the junction leg.
- Similarly the middle-energy leg is fragmented.
- A diquark is formed by combining the flavour and momenta of the two partons left on the low and middle legs closest to the junction point.
- The diquark is connected at one end of the high-energy leg, the junction no longer exists and the string is fragmented via the usual string fragmentation mechanism.

Finding the junction frame for three $\textit{massless partons}$ is trivial, but as soon as one or more of them are massive, the process does not always converge to a stable solution, because such a solution does not exist. (Also in the case where a junction leg has a long chain of gluons, the proper frame can be difficult to find.)

**Fig. 2:**
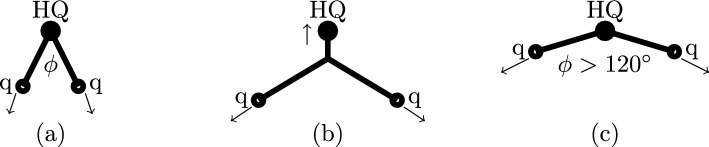
(**a**) A representation of a junction system with a heavy quark (denoted with HQ) and two light partons denoted with (q), (the arrows show their momentum vectors) with an angle $\phi$ between them, (**b**) a junction rest frame system for the same configuration, and (**c**) a scenario where the heavy quark coincides with the junction point and the angle between two light partons is greater than $120^{\circ }$

Figure 2a illustrates a system with a heavy quark and two light colour charges (quarks or gluons) in the initial rest frame of the heavy quark. If the angle $\phi$ between the light charges is smaller than $120^{\circ }$, there is always a frame, in which a junction is at rest as in Fig. 2b. The massive quark moves more slowly, and the corresponding string piece is shorter. For $\phi = 120^{\circ }$ this length goes to zero, and for $\phi > 120^{\circ }$ the junction coincides with the heavy quark, see Fig. 2c. In this case, we find that it is most natural to hadronize the system in the rest frame of the heavy quark. We have implemented this in Pythia8, and one consequence of this new procedure is that the heavy quark is more likely to be the lowest energy leg, and will in addition not be able to fragment into a heavy meson before being joined into a diquark and then ending up in a baryon. (We note that in this situation the Pythia8 fragmentation system instead hadronizes three strings connected at the centre in the rest frame of the whole junction system. This reduces the probability of producing a heavy baryon and overestimates the number of produced hadrons.)

We have made one more change in Pythia8 to enhance the chance of a heavy quark ending up in a baryon, and that is to change the ordering of the junction legs. Instead of taking the leg with the lowest summed energy of the connected partons, we use the sum of absolute spatial momentum instead. In analogy with the change in the $\lambda$-measure in the QCDCR, this will more closely correspond to how long the actual string is, and will more often put the leg with a heavy quark among the two legs that are fragmented first. Again this improves the chances that the heavy quark ends up in a baryon.

Besides changing the actual algorithms in Pythia8 and in the QCDCR model, we have also investigated some of the parameters that can affect the production of charmed baryons. In the diquark formation by combining the two quarks from the low- and middle-energy legs, the spin assignment is done by a set of parameters^3^ suppressing the expected ratio of 3 spin-1 vs. spin-0 states. The default values in Pythia8 are 0.5, 0.7, 0.9, and 1, for the cases where the heaviest quark is u/d, s, c, and b, respectively, but these are not well constrained by experimental data. In the so-called mode-0 tune for the QCDCR model, these were instead all set at 0.0275, which is close to the more well constrained value used for the diquark–anti-diquark breakups in a normal string. There is, however, no reason to expect that these parameters should be the same, since the formation of diquarks in the joining of junction legs is very different from the breakup in strings. And since we know that the QCDCR model has difficulties in describing the production of heavier baryon states, we have checked the effect of raising the values to the default ones in Pythia8 also when using QCDCR.

Since we will here mostly be concerned with charmed baryons that also includes strange quarks, there are also other effects that can influence the production. It is well-known that strangeness enhancement is present not only in heavy-ion collisions but also in high multiplicity $\textrm{pp}$ collisions (see, e.g., [41]). In Lund we have studied the so-called rope hadronisation model [42–44], where overlapping strings gives an increase in the string tension, $\kappa$. This results in an increased probability of strange quarks in the string breakups (c.f., Eq. 1) and the results are promising. Our current implementation does not, however, handle junctions very well, which is why we here have decided to emulate the effect by increasing the overall relative probability of having strange quarks in string breakups^4^ from the default value of 0.217 to 0.4. The number may seem to be high but since most of the charmed baryons are produced at high multiplicities, where there are many dipoles that can reconnect, and hence also many strings can overlap, we do not consider it to be unreasonably high.

The overall charm content in an event is mainly governed by perturbative effects, and can be gauged by the rate of the most common charmed D-mesons, which are reasonably well described by the default Pythia8. With the modifications we have described here, however, a larger fraction of charm quarks will end up in baryons, reducing the rate of D-mesons, and we have therefore decided to compensate for this by increasing the overall charm production by reducing the charm (and bottom) quark mass i Pythia8 from the default value of 1.5 (4.8) GeV to 1.3 (4.2) GeV.

**Fig. 3:**
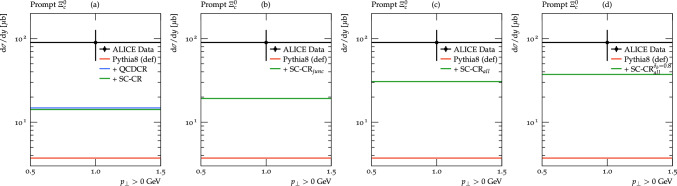
Integrated prompt $\Xi ^0_c$ cross-section for $\textrm{pp}$ at $\sqrt{s} = 5.02$ TeV, for $\mid y\mid < 0.5$ using different options for Pythia8 compared to ALICE data [22]. In all cases, we show Pythia8 default and, from the left, we show (**a**) QCDCR (mode-0), and spatially constrained QCDCR (as SC-CR); (**b**) SC-CR with corrections in the heavy quarks junction formation and fragmentation; (**c**) SC-CR will all the new changes from this work; and (**d**) SC-CR (with all changes) with the $\delta _b$ parameter set to 0.8 fm

To get an indication of the overall effects of the changes we have proposed here, we show in Fig. 3 the rate of direct production of $\Xi ^0_c$ baryons in $\textrm{pp}$ collisions at $\sqrt{s} = 5.02$ TeV. The model results are compared with the ALICE data [22] using the Rivet [45] routine called ALICE_2021_I1863039. In the left-most histogram, we show the results of the default Pythia8 (red line), QCDCR (mode-0) (blue line), and spatially constrained QCDCR (green line). Here we see clearly the effect of introducing the junction reconnections in QCDCR. Our spatially constrained version of the QCDCR gives a slightly reduced rate, mainly because of the constraint, but also because of differently tuned parameters (see [24] for details). In the second to the left histogram, we show the effect of the changes in junction formation and fragmentation for the SC-CR case, and find an increase of around 35%. In the third histogram, we have also added the parameter changes described above and found an additional increase of almost 60%, giving an almost doubled rate compared to the default SC-CR, and a factor 8 more than the default Pythia8. We are, however, still far away from the lower bound of the experimental error bar, and a factor almost three below the central value.

Finally, in the right-most histogram of Fig. 3, we show that if we allow reconnection of dipoles farther separated in the transverse plane by increasing the spatial constraint ($\delta _b$) value in the SC-CR model from 0.5 fm to 0.8 fm on top of the other changes we have made, then we can further enhance the $\Xi ^0_c$ baryon’s production in $\textrm{pp}$ collision events. However, since one of the aims of this paper is to compare to $\textrm{pPb}$ data using the Angantyr model we will in the following keep the tuned value of 0.5 fm, which we have shown in [24] gives a more reasonable description of multiplicities in $\textrm{pPb}$.

## Results

In this section, we want to look more in detail at the effects of the changes we made. We will concentrate on the charmed baryons, but will also look at non-charmed hyperons. We first look at $\textrm{pp}$ collisions to check that we get reasonable results there before we extrapolate the models to $\textrm{pPb}$ collisions using Angantyr.

### Hyperon production in $\textrm{pp}$ collisions

**Fig. 4:**
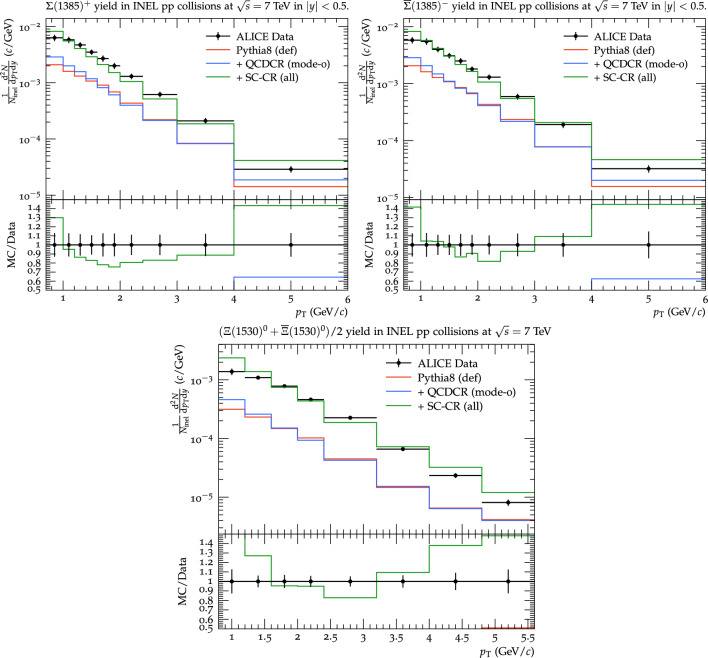
Top: $p_\perp$ differential yield of $\Sigma ^+$ (left) and $\bar{\Sigma }^-$ (right). Bottom: $(\Xi ^0 + \bar{\Xi }^0)/2$ yield as a function of $p_\perp$. The results are from the ALICE experiment [46] for $\textrm{pp}$ collisions at 7 TeV and for mid-rapidity ($\mid$y$\mid$ $< 0.5$). The experimental results are compared with Pythia8 default, QCDCR (mode-0), and spatially constrained QCDCR with all the new changes from this work, which are shown as red, blue, and green lines respectively

Since we have forcibly increased the overall strangeness rate in the Pythia8 string fragmentation, it is important to check that what we have done is not unreasonable. In Fig. 4 we therefore show $p_\perp$ distribution for $\Sigma ^+$, $\bar{\Sigma }^-$, and $(\Xi ^0 + \bar{\Xi }^0)/2$ baryons respectively. The ALICE experiment [46] results for $\textrm{pp}$ collisions at $\sqrt{s}=7$ TeV are used here.^5^ The measurements are reported for inelastic collisions and for the particles in the mid-rapidity region ($\mid$y$\mid$ $< 0.5$).

Comparing the default Pythia8 with and without QCDCR, it is clear that the junction reconnections do not contribute much to strange baryon. Instead, the main production mechanism is diquark breakups in the string fragmentation. We can therefore conclude that the main effect when looking at the changes we have done here is the enhancement of strange (di-)quarks in the string breakups. It can be argued that our enhancement is a bit high, but it is clearly not completely unreasonable.

### Charmed baryon production in $\textrm{pp}$ collisions

We now turn to the charmed baryons and will start with $\Lambda _c$, where we know that the QCDCR model does a reasonable job. Looking back at Fig. 3, we see that our changes increase the $\Xi ^0_c$ rate substantially, and one can fear that this is compensated by a decrease of $\Lambda _c$.

**Fig. 5:**
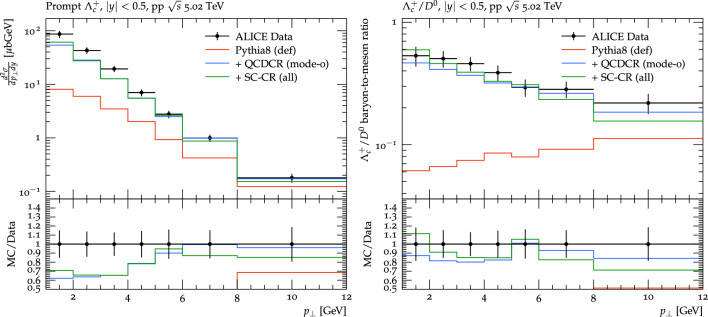
Prompt $\Lambda ^+_c$ distribution as function of $p_\perp$ on the left. The baryon-to-meson ratio for $\Lambda _c^+ /D^0$ as a function of $p_\perp$ on the right. The $\textrm{pp}$ collision at 5.02 TeV results are from the ALICE experiment [12]. The coloured lines represent the same setups as in Fig. 4

To check this, we show in Fig. 5 the $p_\perp$ distribution of the prompt $\Lambda ^+_c$ baryons and $\Lambda _c^+ /D^0$ ratio is compared with the ALICE data [12] (using the same rivet routine as in Fig. 3). Clearly, we maintain a good description of the $\Lambda ^+_c$ cross section and $\Lambda _c^+ /D^0$ ratio, even after all the new changes we have introduced in this work.

We note that the QCDCR, both with and without our changes, gives more enhancement for $\Lambda _c^+$ for low $p_\perp$, as seen both for the yield and for the ratio to the $D^0$ yield. The reason for this is that most strings in an event are fairly parallel to the beam, connecting low-$p_\perp$ partons produced by MPI. So the largest chance to get baryons from junction reconnections is from two or three dipoles from such strings along the beam direction, which then results in low-$p_\perp$ baryons.

The effect is less visible for the strange baryons in Fig. 4 since the relative contribution from junction reconnection is smaller but it is still reflected in a small increase of small $p_\perp$ for QCDCR.

**Fig. 6:**
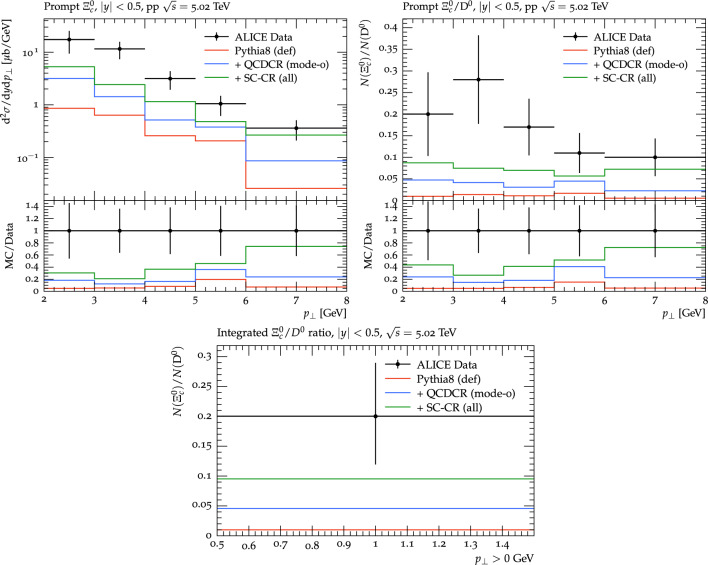
Top left: The prompt $\Xi ^0_c$ cross section as a function of $p_\perp$. Top right: The $\Xi ^0_c /D^0$ ratio as a function of $p_\perp$. Bottom: The integrated $\Xi ^0_c /D^0$ ratio for all $p_\perp >0$. The data is from ALICE experiment [22] $\textrm{pp}$ collisions at $\sqrt{s} = 5.02$ TeV. The coloured lines represent the same setups as in Fig. 4

**Fig. 7:**
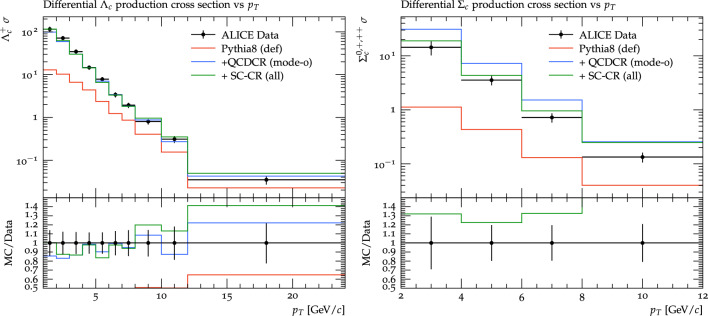
$p_\perp$ differential production cross section of $\Lambda _c^+$ on the left and of $\Sigma _c^{0,+,++}$ on the right. The data is from the ALICE experiment for pp collisions at 13 TeV [14]. The coloured lines represent the same setups as in Fig. 4

**Fig. 8:**
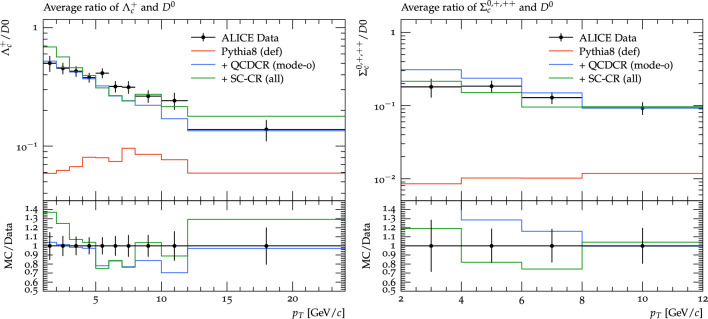
Baryon-to-meson ratio for $\Lambda _c^+ /D^0$ on the left and $\Sigma _c^{0,+,++} /D^0$ on the right. The data is from the ALICE experiment for pp collisions at 13 TeV [14]. The red, blue, and green lines are the same as in the Fig. 7. The coloured lines represent the same setups as in Fig. 4

In Fig. 6, we then show the corresponding comparison for $p_\perp$ distribution of the prompt $\Xi ^0_c$ baryons and the $\Xi ^0_c / D^0$ ratio results obtained at the ALICE experiment [22] for $\textrm{pp}$ collisions at $\sqrt{s}$=5.02 TeV. The cross section distribution basically shows the same thing that we previously showed in Fig. 3, where the overall yield for Pythia8 is far below the data while adding QCDCR brings it closer, and with our changes even more so.

The $\Xi ^0_c / D^0$ ratio is arguably more relevant for assessing our changes, since the overall (perturbatively modelled) charm rate is factored out, and only the change in the non-perturbative modelling is important. Both for the $p_\perp$ distribution and the integrated ratio our changes actually come quite close to the data (note that there is a linear scale for the ratios here). We note that for the $p_\perp$ shape, the data has a tendency to decrease a bit for the lowest $p_\perp$ bin, while the QCDCR model, with and without our changes, seems to continue to rise, mirroring the behaviour in the Fig. 5.

**Fig. 9:**
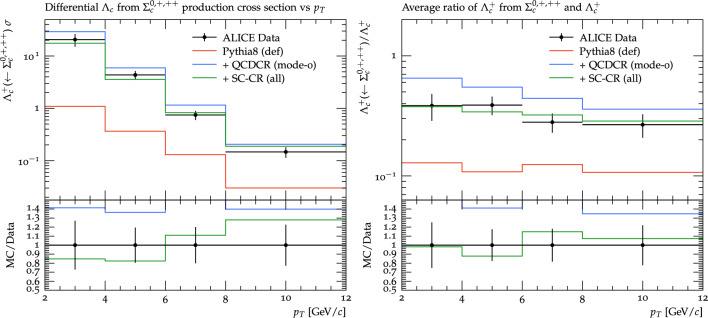
Left: $p_\perp$ differential production cross-section of $\Lambda _c^+$ from $\Sigma _c^{0,+,++}$ decays. Right: Ratio of $\Lambda _c^+$ from $\Sigma _c^{0,+,++}$ decays to the total $\Lambda _c^+$ as a function of $p_\perp$. The data is from the ALICE experiment for pp collisions at 13 TeV [14]

Last year, the ALICE collaboration presented results [14]^6^ also for $\Sigma _c$ baryons in $\textrm{pp}$ collision, this time using data from the LHC run 2 at $\sqrt{s}$= 13 TeV. Figure 7 shows a differential production cross-section for $\Lambda _c^+$ on the left and $\Sigma _c^{0,+,++}$ on the right as a function of $p_\perp$, and in Fig. 8 the same is shown as a ratio to the $D^0$ cross section. We can clearly see that the modification of the QCDCR model done in this paper not only maintains the $\Lambda _c^+$ description but also controls the $\Sigma _c^{0,+,++}$ production rate in Pythia8. Finally in Fig. 9, we show that due to the reduced $\Sigma _c^{0,+,++}$ production cross-section, the fraction of $\Lambda _c^+$ coming from $\Sigma _c^{0,+,++}$ decays, and the ratio to the inclusive $\Lambda _c^+$ both are improved by our modifications to the QCDCR model.

From our changes to the QCDCR, the one mainly influencing the $\Sigma _c$ rate is the change in the parameter controlling the diquark formation in the joining of the smallest junction legs in the fragmentation (see Sect. 3.3.2). Increasing the probability for a charmed diquark to be in a spin-1 rather than a spin-0 state, means that $\Sigma _c^\star$ states are favoured over the $\Sigma _c$ ones in the subsequent fragmentation of the largest leg. As mentioned in Sect. 3.3.2 these parameters were previously completely unconstrained by data and in [16], the authors described the chosen default values as guesswork. In QCDCR (mode-0) the values were set to the same, rather low, value for all quark types, but in our change, we decided to keep the default ones which are higher and dependent on the heavy quark mass. That the probability should be mass dependent is reasonable since the mass splitting between the spin-1 and spin-0 state should be smaller when heavier quarks are involved. (See, e.g., [47] for a discussion on this). Thanks to ALICE we now have data [14] that can actually constrain this parameter. Here also we notice that the $\Lambda _c^+ /D^0$ ratio for low $p_\perp$ is increased.

### $\textrm{pPb}$ collisions

**Fig. 10:**
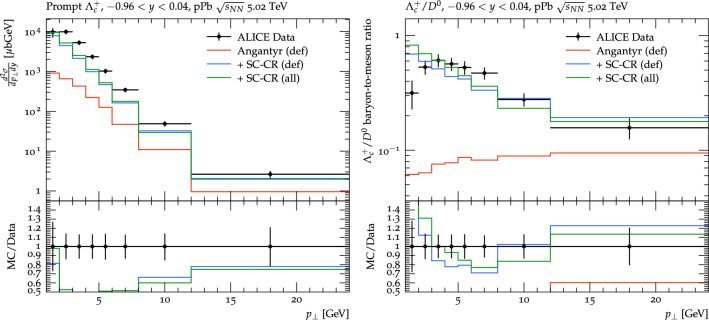
Prompt $\Lambda ^+_c$ distribution as a function of $p_\perp$ on the left. The baryon-to-meson ratio, $\Lambda _c^+ /D^0$, as a function of $p_\perp$ on the right. The red, blue, and green lines are Angantyr default, Angantyr new tune with global CR as SC-CR (def), and the changes we have made in this work in the global CR as SC-CR (all) respectively. The *pPb* collision at 5.02 TeV results are from the ALICE experiment [12]. (Note that the rapidity region, $-0.96<y<0.04$ is given in the collision rest frame, and corresponds to the central, $\mid \eta \mid <0.5$ region in the laboratory frame.)

With a reasonable description of charmed baryon production in $\textrm{pp}$ collisions, we can now use the Angantyr model to extrapolate our results in heavy-ion collisions. In a previous publication, we have shown that the colour reconnection between dipoles in QCDCR can be constrained by introducing a cut in the transverse separation between dipoles. By adjusting the value of this cut we can allow for a global colour reconnection between sub-collisions in heavy-ion collisions and still retain a reasonable description of hadron multiplicities. Since the charmed baryon production has been shown to be a sensitive probe into how the junction reconnections in the model behave, we can now see in more detail if our extrapolation to heavy-ion collisions is reasonable.

In Fig. 10 we show $\textrm{pPb}$ results from the ALICE experiment at $\sqrt{s_{NN}}$= 5.02 TeV for the $\Lambda _c^+$ cross section, and the ratio of this w.r.t. $D^0$ cross section, in comparison with Angantyr model. As expected, the default Angantyr, with colour reconnections only within each sub-collision separately, severely underestimated the rate of $\Lambda _c^+$. Adding our spatially constrained version of the QCDCR model improves the description of data significantly, although the $\Lambda _c$ cross section is still somewhat underestimated. Adding the changes introduced in this paper, however, does not influence the result much. This was to be expected, since also in $\textrm{pp}$ the effect on $\Lambda _c^+$ was minor.

We can see that for low $p_\perp$ the model fails to reproduce the behaviour of the data. This is best seen in the ratio to $D^0$, where our model completely shows no sign of reduction of the ratio at small $p_\perp$. Also this could be expected, as we had also seen indications of this in $\textrm{pp}$ collisions above.

## Conclusion

This paper has shown, for the first time, the effect of applying a modern colour reconnection model to a heavy ion collision, in order to better describe baryon yields. We have shown that the production rates of $\Lambda _c^+$ are dramatically improved in $\textrm{pPb}$ collisions using the QCDCR model, which has previously worked well in $\textrm{pp}$ collisions. We also show that the diquark formation in the joining of the junction legs influences the spin-dependent baryon production, and we require experimental data similar to ALICE [14] to constrain the parameter in Pythia8.

Heavy quarks can only be produced in hard scattering or in a parton shower mechanism in Pythia8. We show that the application of colour algebra in the QCDCR model allows junction formation by connecting three colour dipoles in a junction point. These junctions contribute significantly to baryon production.

We show that for a heavy quark connected to a junction the $\lambda$-measure used in the QCDCR model should be improved. Usually, the $\lambda$-measure calculates the logarithm of the energy of the dipole in the junction rest frame. But if the dipole contains a heavy quark then often the invariant mass of the quark has a non-negligible contribution to the energy of the dipole. Therefore the rapidity span of the heavy quark from the junction point in the junction rest frame should be used as the $\lambda$-measure for such a dipole.

Moreover, when a heavy quark dipole is directly connected to the junction point, the system often fails to obtain a junction rest frame. If the momentum of the heavy quark is low, it is possible that the string piece between the junction and the heavy quark collapses to zero. Thus the heavy quark is directly connected by two strings to the lighter quarks. We show that under such a scenario fragmenting the junction system in the rest frame of the heaviest quark is a good choice.

During the fragmentation of the junction system, the convention is to calculate the energy in every leg and start fragmenting the junction system from the lowest energy leg. Here again, we show that the choice of the new $\lambda$-measure should be the scalar value of the momentum instead of the energy because we should avoid counting the invariant mass of the quarks as the potential energy available in the junction leg.

We notice that apart from the two modifications in the junction formation during CR and junction fragmentation during hadronization, we need strange quarks as many of the heavy baryons contain strange quarks. We have a rope hadronization model, which contributes to the strangeness enhancement in Pythia8. The junction topologies are complex and the string-string interactions in rope hadronization haven’t been implemented for junction configurations. Hence we have compensated it by increasing the string fragmentation probability for the strange quarks.

The charm and bottom quark masses are the other parameters we changed in this paper. To enhance the charm and bottom quark production in the first place we decided to use slightly lower mass values within the proposed mass ranges for the respective quarks.

All these changes together helped us to improve the Pythia8 description for $\Xi$, $\Sigma$, $\Xi _c$, and $\Sigma _c$ baryon production rates. We also managed to keep a good description of the $\Lambda ^+_c$ and $\Lambda ^+_c /D^0$ for different collision energies in $\textrm{pp}$ collisions. For the first time, we show the $\Lambda ^+_c$ and $\Lambda ^+_c /D^0$ results in *pPb* collisions. The results are generated with the global CR in Angantyr and with the changes we introduced in this paper, and they show a visible improvement over the default Angantyr setup.

At this stage, it is also important to note that the increased strange quark fragmentation should be replaced with the appropriate treatment of the rope hadronization model. We may be required to retune some of the parameters because the production of light baryon and other hyperons has not been tracked against the new changes we have made in this work. Moreover, so far we have applied the $\lambda$-measure correction only to the junction formation, but a similar correction should also be applied to the “swing” CR between two dipoles. We hope that the complete treatment of $\lambda$-measure correction will affect the Quarkonium production. Hence exploring the possibility of reproducing Quarkonia suppression in heavy-ion collisions is one of the tasks for future work.

## Data Availability

This manuscript has no associated data or the data will not be deposited. [Authors’ comment: The work done in this paper is a phenomenological modifications in the Pythia/Angantyr model. The experimental data used for the model comparision is referenced appropriately.]

## Footnotes

1 The infrared $\lambda$-measure for a string in Ref. [40], is in the QCDCR model approximated by the sum of these contributions from the individual dipoles.

2 In the junction rest frame, the angles between all momenta of the connected partons are $120^\circ$.

3 The Pythia8 parameter used to set these values is StringFlav:probQQ1toQQ0join.

4 The Pythia8 parameter for this is called StringFlav:probStoUD.

5 The plots were generated using the ALICE_2014_I1300380 routine in Rivet.

6 The analysis is implemented in the Rivet routine ALICE_2022_I1868463.
